# More Than 500 Kids Could Be Saved Each Year! Ten Consensus Actions to Improve Quality of Pediatric Resuscitation in DACH-Countries (Austria, Germany, and Switzerland)

**DOI:** 10.3389/fped.2020.549710

**Published:** 2020-10-07

**Authors:** Philipp Jung, Sebastian Brenner, Iris Bachmann, Christian Both, Francesco Cardona, Christian Dohna-Schwake, Christoph Eich, Frank Eifinger, Ralf Huth, Ellen Heimberg, Bernd Landsleitner, Martin Olivieri, Michael Sasse, Thomas Weisner, Michael Wagner, Gert Warnke, Bernhard Ziegler, Bernd W. Boettiger, Vinay Nadkarni, Florian Hoffmann

**Affiliations:** ^1^University Children's Hospital, University Hospital Schleswig-Holstein, Lübeck, Germany; ^2^Neonatology and Pediatric Intensive Care, University Hospital Carl Gustav Carus, University of Dresden, Dresden, Germany; ^3^University Children's Hospital Zürich, Zurich, Switzerland; ^4^Division of Neonatology, Pediatric Intensive Care and Neuropediatrics, Department of Pediatrics, Medical University of Vienna, Vienna, Austria; ^5^Department of Pediatrics, University Medicine Essen, Essen, Germany; ^6^Department of Anaesthesia, Pediatric Intensive Care and Emergency Medicine, Auf der Bult Children's Hospital, Hanover, Germany; ^7^Children's Hospital, Cologne, Germany; ^8^University Children's Hospital, Mainz, Germany; ^9^Department of Pediatric Cardiology, Pulmology and Intensive Care Medicine, University Children's Hospital, Tuebingen, Germany; ^10^Children's Hospital, Nuremberg, Germany; ^11^Dr. von Hauner University Children's Hospital, Ludwig-Maximilians-University Munich, Munich, Germany; ^12^University Children's Hospital, Medical School Hannover, Hanover, Germany; ^13^University Children's Hospital Graz, Graz, Austria; ^14^University Children's Hospital Salzburg, Salzburg, Austria; ^15^Department of Anaesthesiology and Intensive Care Medicine, Medical Faculty, University Hospital of Cologne, Cologne, Germany; ^16^Children's Hospital of Philadelphia, University of Pennsylvania Perlman School of Medicine, Philadelphia, PA, United States; ^17^Austrian Resuscitation Council, Graz, Austria; ^18^German Resuscitation Council, Ulm, Germany; ^19^Swiss Resuscitation Council, Zurich, Switzerland

**Keywords:** cardiopulmonary resuscitation, children, cardiac arrest, quality improvement, patient safety, pediatric

## Abstract

• Quality and outcome of pediatric resuscitation often does not achieve recommended goals.

• Quality improvement initiatives with the aim of better survival rates and decreased morbidity of resuscitated children are urgently needed.

• These initiatives should include an action framework for a comprehensive, fundamental, and interprofessional reorientation of clinical and organizational structures concerning resuscitation and post-resuscitation care of children.

• The authors of this DACH position statement suggest the implementation of 10 evidence-based actions (for out-of-hospital and in-house cardiac arrests) that should improve survival rates and decrease morbidity of resuscitated children with better neurological outcome and quality of life.

## Introduction

Until recently, circulatory/respiratory arrest in children has received little attention in DACH-countries (Austria, Germany, and Switzerland). Exact numbers of children needing resuscitation are not available for DACH due to insufficient registry and audit infrastructure. When data from North America and Japan are extrapolated, ~5,000 cardiac arrests in children (in- and out of hospital) occur in the DACH-countries per year (1, 2).

Despite a generic trend of improvement in survival rates over time, the overall outcome after cardiac arrest in children is still poor. Many successfully resuscitated pediatric patients have reduced quality of life with persistent impairments in physical, psychological, and executive function, as well as emotional impairment, which are of considerable concern to families and society (3).

Recent studies show that the quality of pediatric resuscitation often does not achieve recommended standards (4–6). Based on substantial international study data, the authors of this DACH position statement suggest the implementation of 10 evidence-based actions (for out-of-hospital and in-house cardiac arrests, OHCA and IHCA) that should improve survival rates and decrease morbidity of resuscitated children with better neurological outcome and quality of life. Due to existing data survival rates with good neurological outcome (PCPC 1 and 2) in OHCA are between 2 and 12%, in IHACA 19–39% (7–10). Depending on that our initiative in actions could improve the rate of survival with good neurological outcome in a range from 10 (OHCA) to 20% (IHCA). This would roughly estimated lead up to a minimum of 500 children/year with better outcomes in DACH-countries, with a total of >35,000 life-years saved.

The following 10 actions/theses—like the “10 Bad Boll Theses for 10,000 lives” (11) published for adult resuscitation—are intended to provide the action framework for a comprehensive, fundamental, and interprofessional reorientation of clinical and organizational structures concerning resuscitation and post resuscitation care of children. The following theses and basic principles are also endorsed by the Austrian, German, and Swiss Resuscitation Councils.

## Actionable Recommendations

### Action/Thesis 1

**Preventing pediatric cardiac arrest has the highest priority**.

The best resuscitation is one that was prevented (12). Although there is contradictory evidence for the benefit of early warning scores (13–15), especially for the reduction of mortality, we call for highlighting this topic with advancing systematic implementation and promoting further development and validation of early warning systems. Medical emergency teams must be formed to take care of these children at risk for resuscitation.

### Action/Thesis 2

**Regular mandatory training in basic life support focusing on adequate chest compressions and ventilation improves patient outcome. Additional short “just-in-time” training sessions can improve the retention of resuscitation skills**.

Pediatric basic life support (PBLS) should be practiced with all staff caring for acute ill pediatric patients in *inter-professional* and *interdisciplinary* training sessions (16–21).

The timely initiation of basic life support and proper performance of high quality CPR including the use of an automated external defibrillator (AED) is a mandatory skill for health care providers. *High quality PBLS* is characterized by correct depth and rate of compression, adequate chest recoil, and sufficient ventilation. Interruptions need to be minimized. Provision of optimal PBLS has been shown to improve the survival of children (22, 23).

During training, participants should receive adequate feedback on performance by real-time audio-visual feedback devices and instructors (24, 25). These instructors should be qualified trainers^*^ [e.g., for internationally approved course formats by the European Resuscitation Council (ERC) or the American Heart Association (AHA)]. Training groups should not exceed eight participants. Although it is unclear what the best training frequency should be, we suggest training sessions *twice annually* and for at least *2 h each*, also because that seems to be convertible. These sessions must be mandatory and offered during ones regular schedule.

Additionally “just-in-time” training may be provided to frontline providers to refresh resuscitation skills and are intended to support regular PBLS trainings. “Rolling refresher” means a manikin is positioned on a cart which is “rolled” directly on to the ward. This training is short (5–10 min) and low-threshold. Content and frequency of training may be adapted to individual needs and have been successfully implemented to improve necessary basic skills or chest compression quality in particular (26–29).

### Action/Thesis 3

**Medical staff working with acutely ill infants should receive appropriate pediatric life support training (e.g., EPALS, PALS, PEARS, EPILS, etc., according to their role). This training must include aspects of crew-resource management. Further research on the most effective mechanisms to provide training is needed**.

All medical staff working in high-risk areas (*intensive care units, high dependency units*, and *pediatric emergency rooms)* should receive appropriate pediatric advanced life support training titrated to their scope of practice. Internationally approved course formats are provided by the European Resuscitation Council (ERC) [EPALS] or by the American Heart Association (AHA) [PEARS, PALS].

These formats extend training to advanced issues during resuscitation, and also include team aspects such as teamwork and leadership, task management, timely decision making, as well as situation awareness. They also teach adequate provision of care to critically ill patients (preventing resuscitation) as outlined by current ILCOR resuscitation guidelines.

Recent initiatives to improve resuscitation efforts in pediatrics have focused on the implementation of action-linked phrases, cognitive aids, rapid cycle deliberate practice, CPR coaches, comprehensive debriefing, and simulation training (29). Reflection on current practice and research to provide the most effective way of optimize resuscitation efforts and how to train staff are urgently needed.

### Action/Thesis 4

**The use of objective (e.g., live feedback systems) and subjective feedback (e.g., CPR-coach) optimizes the quality of chest compressions and should be used in both, training as well as in daily prehospital and clinical work**.

Quality of basic life support skills, especially chest compressions, decisively impacts patient outcome and can be optimized by using live feedback systems (30, 31). Feedback systems approved for pediatric patients should be routinely used. They can increase chest compression quality by offering real-time information about compression rate, compression depth, and leaning force. They should be routinely used in training sessions as well as in daily in- and out-of-hospital routine. Therefore, they should be cost-effective and easy to use.

However, during both real and simulated cardiac arrests, providers often deliver poor CPR despite receiving visual (and sometimes verbal) feedback from the defibrillator. A CPR coach is a trained person who provides real-time verbal feedback of CPR performance and improves compliance to CPR guidelines. This also supports the resuscitation leader so she/he can focus on other aspects during resuscitation. In the presence of CPR feedback technology, the addition of a trained CPR coach into resuscitation teams adds to CPR quality metrics which in turn are associated with improved survival outcomes from pediatric cardiac arrest (32–34).

### Action/Thesis 5

**Structured debriefings of resuscitations lead to improvements in care and outcomes of resuscitated children**.

Structured debriefings of emergency situations are already recommended by the ERC and other resuscitation societies. Debriefings can take place immediately (“hot”) and/or after a certain period of time (“cold”) after the resuscitation event. Ideally, they should include at least the team members involved in the code. Participation in debriefings may also include non-involved team members as these debriefings are a valuable learning opportunity. Debriefings have a positive impact on team performance and employee satisfaction, leading to an increase of the quality of care. Furthermore, they increase survival rates of resuscitated children (35, 36).

### Action/Thesis 6

**Post-resuscitation treatment is critical to improve outcome of resuscitated children and should follow standardized protocols. The aim is to set up specialized centers for pediatric post-resuscitation care**.

As in adults, post-resuscitation treatment and the level of care for children after cardiac arrest impacts outcome (37). Treatment includes targeted temperature management, lung-protective ventilation achieving normoxia, and the prevention of hypocapnia. Furthermore, managing blood pressure to provide adequate cerebral perfusion, avoid burden of hypotension (38), avoiding disturbances of blood glucose and electrolytes, administering adequate analgesia and sedation, as well as monitoring to detect and treat status epilepticus are mandatory. Care also extends to the family of the patient and initiation of early rehabilitation of pediatric patients after resuscitation (39–42). This level of care is not available in every pediatric department. Therefore approved “Pediatric Cardiac Arrest Centers,” either physical or virtual, should be established correspondingly to post-resuscitation care centers in adults (43). These centers may also support less specialized units (e.g., by telemedicine) in initial stabilization or allow transfer of these children to these centers.

### Action/Thesis 7

**Basic data of every resuscitated child should be entered into an audit or registry. Registry participation should be obligatory for every hospital and EMS system, and should include preclinical, in-hospital, and post-hospital outcome data**.

Although a resuscitation registry currently exists in Germany, very little data of resuscitated children in the DACH region are recorded. Establishing a registry that collects preclinical, in-hospital, and post-hospital outcome (neurological and in general) data from every pediatric resuscitation is essential to guarantee quality improvement. As existing data bases are still voluntary, steps must be taken to ensure every hospital treating pediatric patients participates in either a local, European, or worldwide registry (e.g., Pediatric Resuscitation Quality Collaborative, PediResQ, https://www.pedires-q.org) (44, 45).

A template for a successful registry is the TraumaRegister® of the German Trauma Society (DGU, http://www.traumaregister-dgu.de/index.php?id=144), which has set standards worldwide for the quality management of seriously injured patients (46). At present, it involves nine countries with over 700 participating hospitals and more than 270,000 documented cases since 1993.

### Action/Thesis 8

**Children's hospitals require regular exchange and networking to deliver better quality of care and improve quality**.

In addition to post-resuscitation therapy, successfully resuscitated children often require treatment from several pediatric sub-specialties (e.g., pediatric intensive care, neurology, cardiology, infectious disease, radiology, psychology, and rehabilitation). However, only few children's hospitals can offer all of these services. Despite of a centralization in post-resuscitation care the implementation of telemedical and other network structures are needed to provide comprehensive health care to ensure quality and efficiency. As in Neonatology certain parameters should be identified that reflect quality of care (47). These parameters should allow a comparability of care and identify further research and education topics.

### Action/Thesis 9

**Every children's hospital should have medical and nursing supervisors for clinical resuscitation who are responsible for implementing adequate on-site and ongoing pediatric resuscitation services and training**.

The quality of resuscitation after IHCA and related outcomes vary greatly from hospital to hospital and also from time of day (48, 49). In order to achieve the best possible result from resuscitation, a dedicated contact person is necessary. This “resuscitation supervisor” should be established in every children's hospital.

The primary tasks of the supervisor are the implementation, organization, education, and monitoring of in-hospital resuscitation training and data management. In addition, this person should be the central point of contact for all matters related to pediatric resuscitation in the hospital and the preferred contact for other external hospitals or emergency services. The resuscitation supervisor has to be supported effectively by the hospital management in terms of time, logistics, and administration.

### Action/Thesis 10

**Hospitals should aim to implement and integrate** all recommendations above **to achieve improvements in pediatric resuscitation**.

In order to improve the outcome of critically ill children in the long-term, a comprehensive and unified system concept is needed. Ideally, *all* of the above actions should be considered and implemented as a complete package.

However, it may be more feasible to start with few elements first and aim to implement the others stepwise until the complete proposed bundle is put into practice.

## Conclusions

Physical and mental disability resulting from a child's cardiac arrest is of exceptional family and societal importance. The total number of pediatric cardiac arrest resuscitations is much lower (10%) compared to adults. However, if outcomes are calculated in terms of quality of life years saved, the impact has high economic relevance.

With these 10 actions/theses we want to focus the broad field of topics concerning pediatric resuscitation. It all starts with the importance of improving the prevention and detection of life-threatening events in children, in order to avoid pediatric cardiac arrest. When resuscitation is necessary, it can only be effective if consistent and effective training is consistently offered and titrated to the appropriate scope of advanced pediatric care.

Learning from every patient is most important. The quality of resuscitation and post-resuscitation care must be measured and evaluated, and this data has to be used to identify challenges to optimize care. A structured collection of treatments and outcome data in a centralized mandatory audit/registry offers the possibility of identifying in- and out-of-hospital care strengths (what went well and why) and challenges and creates roadmaps for quality improvement. It has to be possible that short and long-term outcomes are linked with the initial resuscitation parameters to learn and show how the early performance impacts the later outcome.

Our goal is to improve the outcome after pediatric cardiac arrest in the DACH-region and around the world. We feel confident that the implementation of these 10 actions/theses will improve current care and be of great benefit for all.

## Author Contributions

All authors conceived and designed the consensus of the 10 actions for improvement of pediatric CPR. All authors performed substantial revisions of this article. All authors gave their final approval of the version to be published.

## Conflict of Interest

The authors declare that the research was conducted in the absence of any commercial or financial relationships that could be construed as a potential conflict of interest.
